# Drivers of zoonotic *Campylobacter* Species transmission in slaughterhouse settings: Insights from Nigeria for global One Health improvement

**DOI:** 10.1016/j.onehlt.2026.101371

**Published:** 2026-02-20

**Authors:** Emmanuel O. Njoga, Kennedy F. Chah, James W. Oguttu

**Affiliations:** a Department of Agriculture and Animal Health, College of Agriculture and Environmental Sciences, University of South Africa, Science Campus, Florida, Johannesburg, South Africa; b Department of Veterinary Public Health and Preventive Medicine, Faculty of Veterinary Medicine, University of Nigeria, Nsukka 410001, Nigeria; c Department of Veterinary Microbiology and Immunology, Faculty of Veterinary Medicine, University of Nigeria, Nsukka 410001, Nigeria

**Keywords:** *Campylobacter* infections, Slaughterhouse practices, Occupational exposure, Zoonotic risk, Food safety

## Abstract

Slaughterhouse workers (SHWs) face occupational risk of zoonotic *Campylobacter* infection (ZCI) but exposure data are limited in low- and middle-income countries (LMICs), particularly Nigeria. This study assessed behavioral, educational, and infrastructural factors affecting *Campylobacter* exposure among 188 SHWs in Enugu, Nigeria. Data on socio-demographics, hygiene practices, knowledge of zoonotic transmission, and self-reported gastroenteritis symptoms were collected via a validated questionnaire. Descriptive statistics and multivariable logistic regression identified determinants of risky practices, knowledge gaps, and symptoms. Overall, 56.4% of SHWs were classified as high-risk for *Campylobacter* exposure. Unsafe practices included non-use of personal protective equipment (PPE) (77.1%), washing multiple carcasses in the same bowl (54.8%), eating or drinking during processing (45.7%), and consuming raw or undercooked meat (36.7%). Non-potable water was used by 90.4% of respondents, with only 14.1% purifying it. Behavioral practices aiding ZCI were significantly associated with lack of hygiene training (OR = 3.7, 95% CI: 1.7–8.0, *p* = 0.001), low educational attainment (OR = 2.8, 95% CI: 0.15–0.85, *p* = 0.020), age ≥ 45 years (OR = 2.1, 95% CI: 0.25–0.92, *p* = 0.028), and urban slaughterhouse location (OR = 2.4, 95% CI: 1.26–4.57, *p* = 0.007). Knowledge gaps were common, with 28–57% unaware of transmission risks via meat, water, PPE, or eating during processing. Self-reported gastroenteritis symptoms were documented in 64.4% of SHWs, with 76% continuing work while symptomatic. Having <10 years' work experience (AOR = 2.84, 95% CI: 1.36–5.95, *p* = 0.006), lack of training (AOR = 2.74, 95% CI: 1.12–6.67, *p* = 0.027), low knowledge of *Campylobacter* transmission dynamics (AOR = 4.46, 95% CI: 2.02–9.87, *p* < 0.001), and high-risk practices (AOR = 6.98, 95% CI: 3.0–16.2, p < 0.001) were independently associated with symptoms. These findings highlight critical occupational exposure, entrenched unsafe practices, and knowledge deficits. Targeted One Health interventions, including provision of potable water, mandatory use of PPE, and context-specific hygiene training are urgently needed to reduce zoonotic *Campylobacter* transmission risks and enhance food safety in the study area.

## Graphical abstract

**Figure:**
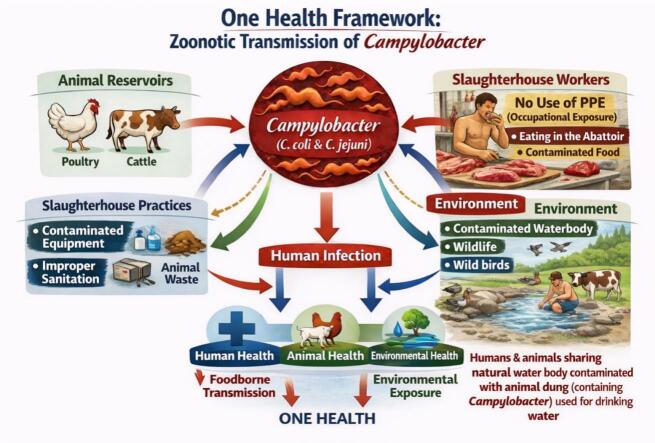

## 1 Introduction

The occurrence of zoonotic pathogens in the slaughterhouse settings continues to threaten food safety and human health, with *Campylobacter* species predominating as the cause of foodborne gastroenteritis and diarrheal diseases globally. Annually, *Campylobacter jejuni* and *Campylobacter coli* collectively account for an estimated 500 million cases of gastroenteritis disorders worldwide [1]. The burden is disproportionately concentrated in low- and middle-income countries (LMICs), where inadequate food safety infrastructure and limited access to safe drinking water exacerbate risks of transmission [2], [3], [4]. In these countries, factors like poor slaughterhouse practices, reliance on contaminated water sources, inadequate sanitation infrastructure and practices, and contamination of fresh foods, especially animal products, increase the risk of zoonotic *Campylobacter* transmission [4], [5], [6], [7]. These risks are excessively borne by vulnerable populations, particularly children, the elderly and immune compromised individuals [4], [5], [6], [7].

*Campylobacter* transmission typically occurs through the consumption of contaminated animal-derived products, direct contact with infected animals, or exposure to contaminated environments, making slaughterhouses a critical point for amplification and dissemination of the zoonotic pathogen [8], [9], [10], [11], [12], [13]. Unlike other foodborne pathogens, *Campylobacter* exhibits a remarkably low infectious dose, estimated at fewer than 500 colony-forming units, which facilitates rapid zoonotic transmission even at minimal exposure [14], [15], [16]. The public health burden is compounded by its association with severe sequelae such as Guillain-Barré syndrome, reactive arthritis, and irritable bowel syndrome [17], [18], [19]. The WHO designates *Campylobacter* spp. as “a priority pathogen requiring urgent new antibiotics”, underscoring the need for integrated One Health interventions beyond conventional sectorial approaches [20], [21].

Slaughterhouses are critical nodes in the food-processing value chain where humans, livestock, and the environment intersect, creating optimal conditions for cross-species transmission of pathogens [12], [13], [22], [23]. Unhygienic meat handling practices during carcass processing and limited awareness of zoonotic risks among slaughterhouse workers (SHWs) can facilitate the persistence and spread of *Campylobacter* within and beyond abattoir environments [24], [25]. Meat contamination with zoonotic pathogens, particularly *Campylobacter*, can occur at multiple stages of slaughterhouse operations, spanning from lairage and evisceration to downstream carcass processing, with faecal materials, animal hides, processing water, and contact surfaces serving as critical reservoirs and vehicles of transmission [26], [27]. In many LMICs, including Nigeria, slaughterhouse operations are often informal, minimally regulated, and poorly resourced, which heightens the likelihood of human exposure to zoonotic pathogens and dissemination into the food supply chain [5], [12]. Human exposure to zoonotic pathogens in slaughterhouse settings, and their subsequent dissemination through the food chain, is exacerbated by inadequate training of workers, weak enforcement of food safety regulations, and entrenched cultural practices that normalize unsafe handling of animal products [12]. These problems not only increase the risk of human foodborne illnesses but also endanger occupational health, perpetuate disease transmission in food-producing animals, and exacerbate environmental contamination through improper waste disposal.

The One Health problems associated with *Campylobacter* are enormous. Beyond being the leading cause of bacterial foodborne gastroenteritis in humans, *Campylobacter jejuni* and *C. fetus* subsp. *venerealis* are also implicated in infertility and abortions in small ruminants and cattle, respectively, resulting in significant economic losses in livestock production [28], [29], [30]. Furthermore, the ability of *Campylobacter* to acquire and disseminate antimicrobial resistance determinants, particularly against fluoroquinolones and macrolides, the first-line antimicrobials for treatment, poses a serious global threat [10], [31]. Environmental contamination from slaughterhouse effluents sustains zoonotic pathogen transmission cycles by polluting water and soils, thereby facilitating the spread to livestock and humans. The interconnectedness of these risks illustrates the quintessential One Health challenge, where interventions must integrate veterinary, medical, and environmental perspectives to achieve sustainable outcomes. By identifying slaughterhouse practices and knowledge gaps in zoonotic *Campylobacter* transmission, this study generates first-hand evidence that is crucial for informing targeted local interventions as well as guiding international policy development on *Campylobacter* control.

Given the substantial economic and public health burden of foodborne *Campylobacter* infections and the paucity of data on risk practices and knowledge gaps in Enugu State slaughterhouses, despite reports of human and animal infections [8], [9], [10], this study investigates these factors as a case study with broader implications for One Health, globally. Specifically, it seeks to characterize the behavioral and operational practices that facilitate pathogen exposure, assess the awareness and perceptions of SHWs regarding zoonotic risks, and identify opportunities for targeted interventions. Nigeria provides a particularly valuable setting for such a study given its expanding livestock sector, widespread informal slaughter facilities, and high burden of foodborne diseases [32]. Findings from this study are critical to informing evidence-based interventions that support policy formulation, capacity building, and integrated health promotion strategies, not only in Nigeria but also in other LMICs facing similar One Health challenges.

## 2 Materials and methods

### 2.1 Ethical approval and informed consent

Ethical approval for this study was obtained from the Research Ethics Committee of the Enugu State Ministry of Health (Reference No. MH/MSD/REC21/232). Prior to participation, all respondents were provided with comprehensive information regarding the study objectives, procedures, potential risks, and benefits. Oral informed consent was obtained from each participant, and strict measures were implemented to ensure the confidentiality and anonymity of all responses. Personal identifiers, including residential addresses and telephone numbers, were not collected to protect participant privacy, in full compliance with internationally recognized ethical standards for human research.

### 2.2 Study area

The study was carried out in Enugu State, South-eastern Nigeria, as detailed in previous reports [33], [34], [35], [36]. The state is made up of three senatorial zones. Enugu State is situated in the Southeast geopolitical zone of Nigeria, positioned at latitude 6°27′10″ N and longitude 7°30′40″ E. The population is predominantly composed of civil servants, traders, and subsistence farmers, although food-animal production is widely practiced as a supplementary source of income. Cattle, poultry, and pigs are the primary food-producing animals processed in most slaughterhouses in Enugu State, reflecting the dominance of beef, chicken, and pork as the major meat types consumed.

Ecologically, Enugu State lies within the derived savannah–tropical rainforest transition zone and is characterized by favourable year-round climatic conditions that enable microbial pathogens to thrive. The state experiences peak average daily temperatures of about 36 °C in February–March and lows of approximately 16 °C in November–December. These fluctuations occur alongside high relative humidity (60–80%) and a bimodal rainfall pattern exceeding 1500 mm annually.

### 2.3 Selection of slaughterhouses

**Fig. 1:**
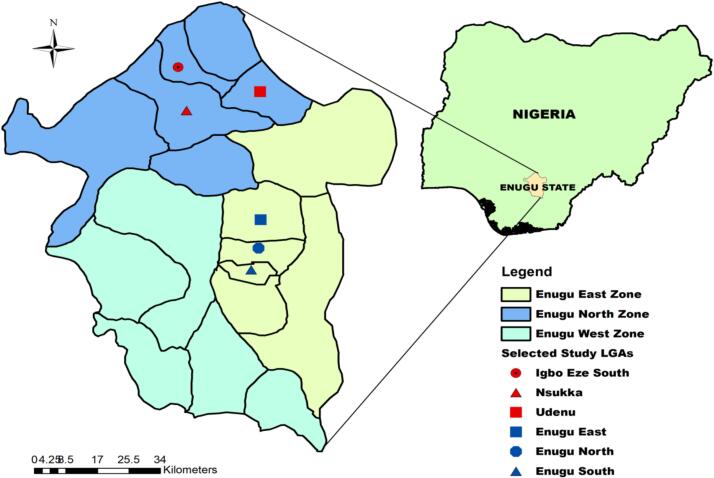
Enugu State in Nigeria, showing the senatorial zones and local government areas where the selected slaughterhouses surveyed are located.

This study was conducted across six major slaughterhouses and slaughter slabs located in Enugu State, Nigeria. Two of the three senatorial zones, Enugu North and Enugu East, in the state were purposively selected based on the availability of slaughter facilities. Enugu South senatorial zone that is largely an agrarian settlement was not selected. Within each selected senatorial zone, three slaughter facilities processing either cattle or poultry carcasses were further purposively selected. The inclusion criteria included high workforce density, substantial slaughter throughput, and strategic representation of both urban and rural contexts to ensure variability in processing practices and associated zoonotic risk profiles. The six facilities ultimately selected were Orie-Igbo-eze, Ikpa, Orba, Mami, Akwata, and Artisan. The geographic distribution of these facilities across the corresponding Local Government Areas (LGAs) within the two senatorial zones is illustrated in Fig. 1.

### 2.4 Instrument of data collection - questionnaire

A questionnaire consisting of 29 items, organized into four sections, was administered during the survey to collect data using structured interviews. The instrument was designed to obtain information on: (1) socio-economic and demographic characteristics (age, gender, marital status, educational status, job description, work experience, and training on hygienic/modern processing methods); (2) involvement in risk practices with potential to facilitate the acquisition and spread of *Campylobacter* infection in slaughterhouse settings; (3) occurrence of gastroenteritis symptoms among SHWs; and (4) knowledge of the workers regarding modes of zoonotic transmission of *Campylobacter* organisms during slaughterhouse operations.

### 2.5 Questionnaire validation

The questionnaire was first subjected to face and content validation following the procedures described by Bolarinwa [37]. Subsequently, a six-member panel of veterinary public health experts independently reviewed the instrument. Each of the 29 items was assessed for relevance and clarity on a 4-point ordinal scale, and recommendations were made accordingly. Based on the expert scores, the scale-level content validity index (s-CVI) and the mean item-level content validity index (mean i-CVI) were calculated as documented by previous reports [38], [39], [40], [41]. Items with s-CVI or i-CVI values below 0.90 were revised to improve clarity and relevance, after which the indices were recomputed. The criteria for scoring each domain were as follows:

(a) Relevance**:** 1 = not relevant; 2 = relevant but requires major revision; 3 = relevant but requires minor revision; 4 = very relevant, no revision required.
(b) Clarity**:** 1 = not clear; 2 = clear but requires major revision; 3 = clear but requires minor revision; 4 = very clear, no revision required.

The revised questionnaire was piloted on 20 respondents outside the study area. Feedback from the pilot study informed further refinements, though the responses from the pilot study were excluded from the main dataset. Reliability of the instrument was determined using Cronbach's alpha, which yielded a coefficient of 0.88, exceeding the 0.7 benchmark, thus confirming internal consistency.

### 2.6 Study design and selection of respondents

The study employed a cross-sectional design using a questionnaire to assess risk practices and knowledge gaps that facilitate the zoonotic transmission of *Campylobacter* species during routine slaughterhouse operations. A total of 211 SHWs were initially selected through simple random sampling (SRS) from a sampling frame of 386 eligible workers. The sampling frame was constructed based on a headcount of the workers who reported for duty at the six selected slaughterhouses on the day of the survey. The SRS technique involved tossing a coin, wherein the appearance of the head indicated inclusion and the tail indicated exclusion from the sample. Although 211 workers were randomly selected, only 188 ultimately participated in the study, as 23 declined to respond due to time constraints and concerns about interference with their routine carcass processing activities.

### 2.7 Questionnaire survey procedure

The validated questionnaire was administered once at each of the six selected slaughterhouses but on different days to all 188 participants who consented to partake in the study. The survey was conducted through interview schedules, consistent with the ethical principles of the Declaration of Helsinki [42]. For respondents with limited proficiency in English, the questions were interpreted in the Igbo, the local dialect, most commonly used in the study area to ensure comprehension. Extreme care was taken to guarantee that each worker participated only once.

Risk of *Campylobacter* infection was assessed using six structured items that captured practices within the slaughterhouse environment known to increase exposure risk. Each correct response was awarded one point, while incorrect responses scored zero, giving a maximum obtainable score of six points. The cumulative scores yielded a mean risk score of 2.8 ± 1.2. Based on this mean, respondents were categorized into two groups: low risk (< 3 points) and high risk (≥ 3 points). Similarly, knowledge of *Campylobacter* transmission during carcass processing was evaluated using ten items. Each correct response attracted one point, with a maximum possible score of ten. The summed knowledge scores produced a mean of 5.6 ± 1.8. This mean was then used as a cutoff to classify respondents into two groups: adequate knowledge (≥ 6 points) and inadequate knowledge (< 6 points).

### 2.8 Data analysis and presentation

Completed questionnaires (*n* = 188) were reviewed for completeness, coded, and entered into Microsoft Excel before export to GraphPad Prism version 8.0.2 (GraphPad Inc., San Diego, CA, USA) for analysis. Descriptive statistics were computed for all variables and presented as frequencies and percentages in Tables. The outcome variable, self-reported gastroenteritis symptoms, was coded as 1 = Yes and 0 = No.

Associations between slaughterhouse workers' socio-demographic characteristics, hygiene practices, occupational exposures, and gastroenteritis symptoms were initially assessed using bivariate binary logistic regression, with each predictor entered independently. Crude odds ratios (ORs) with 95% confidence intervals (CIs) and corresponding *p*-values were computed. Variables with *p* ≤ 0.20 at the bivariate level were considered for inclusion in the multivariable logistic regression model. Multicollinearity among predictors was assessed prior to model fitting. Adjusted odds ratios (AORs) with 95% CIs were estimated to identify factors independently associated with gastroenteritis symptoms. Statistical significance was set at *p* < 0.05 for all analyses.

## 3 Results

### 3.1 Socioeconomic characteristics of slaughterhouse workers

A total of 188 SHWs participated in the questionnaire survey. The majority were male (91.5%, 172/188), and more than half (57.4%, 108/188) were younger than 45 years (Table 1). Of the respondents, 84 (44.7%) were primarily involved in cattle carcass processing, whereas 104 (55.3%) processed poultry carcasses. Only 51 respondents (27.1%) had attained tertiary education. Although 91 workers (48.4%) reported ≥10 years of working experience, only 47 (25.0%) had received formal training on hygienic carcass or meat processing. Additional socio-demographic characteristics of the respondents are summarized in Table 1.

**Table 1:** Socioeconomic characteristics of SHWs (*n* = 188) surveyed in Enugu State.

Variable	Frequency	Proportion (%)
Gender		
Male	172	91.5
Female	16	8.5
Age Category		
Less than 45 years	108	57.4
≥ 45 years	80	42.6
Marital status		
Single	111	59.0
Married	77	41.0
Occupation		
Poultry carcass processor	104	55.3
Cattle carcass processor	84	44.7
Location		
Nsukka (Semi-urban/rural)	97	51.6
Enugu (Urban)	91	48.4
Carcass/meat processing experience		
Less than 10 years	97	51.6
≥ 10 years	91	48.4
Highest educational level attained		
Tertiary education	51	27.1
No tertiary education	137	72.9
Have had formal training on hygienic carcass/meat processing		
Yes	47	25
No	141	75

### 3.2 Risk practices with potential to heighten the risk of *Campylobacter* transmission during meat processing

A total of 82 (43.6%) workers had mean risk score of <3 and therefore classified as having low exposure risk, whereas 106 (56.4%) were categorized as high risk (mean risk score ≥ 3) for *Campylobacter* infection. The predominant risk practices that could facilitate the acquisition and spread of zoonotic *Campylobacter* during carcass and meat processing included: failure to use personal protective equipment (77.1%, 145/188); washing multiple carcasses in the same bowl of water (54.8%, 103/188); eating or drinking while processing carcasses (45.7%, 86/188); and consumption of raw or undercooked meat (36.7%, 69/188). Comprehensive details on the involvement of SHWs in these risk practices are presented in Table 2.

**Table 2:** Involvement of SHWs (*n* = 188) in risk practices that aid acquisition and spread of *Campylobacter* species infections during carcass/meat processing in Enugu State, Nigeria.

Variable	Frequency	Proportion (%)
Water types used for carcass processing		
Well water	45	23.9
Borehole water	72	38.3
Potable water (pipe borne water)	18	9.6
Natural water bodies (streams and rivers)	15	8.0
Rain water	38	20.2
If no access to potable water, is water sanitized with sanitizer before use?		
Yes	24	14.1
No	146	85.9
Do you use the same bowl of water to wash more than one carcass during the processing?		
Yes	103	54.8
No	85	45.2
Do you cleanly wash-off meat cutting surfaces and rinse your knife before processing the next carcass?		
Yes	86	45.7
No	102	54.3
Do you eat or drink while processing carcasses?		
Yes	107	56.9
No	81	43.1
If yes, do you wash your hands with soap and running water before eating? (*n* = 107)		
Yes	55	51.4
No	52	48.6
Do you use personal protective equipment (PPE) while processing carcasses?		
Yes	43	22.9
No	145	77.1
If you use PPE, do you clean and disinfect them daily after your routine duties? (*n* = 43)		
Yes	17	39.5
No	26	60.5
Do you consume raw or undercooked meat during carcass processing?		
Yes	69	36.7
No	119	63.3
Have experienced gastroenteritis symptoms (diarrhoea, dysentery, abdominal cramps, etc.) 90 days prior to the survey date?		
Yes	121	64.4
No	67	35.6
If yes, did you process carcasses/meats for human consumption while having the gastroenteritis symptoms? (*n* = 121)		
Yes	92	76
No	29	24

The proportions of water types used for processing carcases and the respondents involved were as follows: well water (23.9%, 45/188), borehole water (38.3%, 72/188), natural water bodies (8%, 15/188), rain-harvested water (20.2%, 28/188) and potable (pipe-borne) water (9.6%, 18/188). Of the 170 (90.4%) SHWs that used non-potable water for carcass/meat processing, only 24 (14.1%) respondents purified the water with water sanitizer before the use (Table 2).

Determinants of exposure to *Campylobacter* species infections among the carcass processors were age (OR = 2; p = 0.028), location (OR = 2; p = 0.007), training on hygienic carcass processing (OR = 3.7; *p* = 0.001) and educational level (OR = 3.7; *p* = 0.020) (Table 3).

**Table 3:** Association between socioeconomic variables and risk levels of *Campylobacter* infection among carcass processors (*n* = 188) surveyed in Enugu State, Nigeria.

Variable	Category	Number of Low Risk Respondents (%)	Number of High Risk Respondents (%)	OR (95% CI)	p-value
Gender	Male	76 (44.2)	96 (55.8)	1 (Ref)	–
	Female	6 (37.5)	10 (62.5)	0.575 (0.452–1.374)	0.318
Age category	< 45 years	41 (38.0)	67 (62.0)	1 (Ref)	–
	≥ 45 years	41 (51.2)	39 (48.8)	2.096 (0.246–0.924)	0.028*
Occupation	Poultry carcass processor	43 (41.3)	61 (58.7)	1 (Ref)	–
	Cattle carcass processor	39 (46.4)	45 (53.6)	0.940 (0.488–1.810)	0.853
Location	Nsukka (peri-urban/rural)	51 (52.6)	46 (47.4)	1 (Ref)	–
	Enugu (urban)	31 (34.1)	60 (65.9)	2.404 (1.264–4.571)	0.007*
Work experience	< 10 years	32 (42.1)	44 (57.9)	1 (Ref)	–
	≥ 10 years	50 (44.6)	62 (55.4)	0.846 (0.442–1.620)	0.615
Training on hygienic carcass processing	Yes	28 (59.6)	19 (40.4)	1 (Ref)	–
	No	54 (38.3)	87 (61.7)	3.738 (1.748–7.995)	0.001*
Education	Tertiary	27 (52.9)	24 (47.1)	1 (Ref)	–
	No tertiary	55 (40.1)	82 (59.9)	2.817 (0.149–0.846)	0.020*

OR = Odds Ratio; CI = Confidence Interval; Ref = Reference category, *p < 0.05 indicates statistically significant association.

### 3.3 Knowledge of modes of zoonotic transmission of *Campylobacter* species

The mean knowledge score among SHWs was 5.8 ± 1.8. Based on the predetermined cut-off, 88 (46.8%) respondents demonstrated adequate knowledge (mean score ≥ 6), whereas 100 (53.2%) exhibited inadequate knowledge (mean score < 6). Notably, only 43.1% (81/188) of SHWs were aware that *Campylobacter* infection in food-producing animals (FPAs) can be transmitted to humans (Table 4). Similarly, significant knowledge gaps were observed regarding specific transmission routes: 28.2% (53/188), 51.1% (96/188); 52.7% (99/188), 56.9% (107/188), and of slaughterhouse respectively, were not aware that *Campylobacter* infection can result from (i) consumption of contaminated raw or undercooked meat, (ii) eating or drinking during carcass processing activities (iii) the use of contaminated water during carcass processing, and (iv) failure to use personal protective equipment (PPE) while dressing carcasses (Table 4).

**Table 4:** Knowledge of modes of transmission of *Campylobacter* infection among SHWs (*n* = 188) in Enugu State, Nigeria.

Variable	Frequency	Proportion (%)
Food-producing animals can harbour harmful bacteria like *Campylobacter* species		
Yes	124	66
No	64	34
Can *Campylobacter* infection in food-producing animals spread to humans?		
Yes	81	43.1
No	107	56.9
Can human *Campylobacter* infection result from consumption of contaminated raw/undercooked meat?		
Yes	135	71.8
No	53	28.2
Can use of contaminated water for carcass processing during slaughterhouse operations aid human *Campylobacter* infection?		
Yes	89	47.3
No	99	52.7
Non-use of PPE can enhance zoonotic *Campylobacter* infection among occupationally exposed people, particularly SHWs?		
Yes	81	43.1
No	107	56.9
Eating and or drinking while processing carcass, especially with unwashed hands, may increase the chances of zoonotic *Campylobacter* infection among SHWs		
Yes	92	48.9
No	96	51.1
Can human *Campylobacter* infection lead to other serious health complication		
Yes	63	33.5
No	125	66.5
*Campylobacter* species from gut contents of slaughtered food-producing animals can contaminate drinking water sources and vegetable when untreated abattoir wastes/effluents are washed into natural water bodies or used to fertilize farms		
Yes	163	86.7
No	7	3.7
I do not know	18	9.6
*Campylobacter* species infections can spread by feeding untreated gut contents to pigs or using it to fertilize fish ponds		
Yes	113	60.1
No	48	25.5
I do not know	27	14.4
Handling eviscerated foetuses with bare hands or feeding the raw meat to dogs can also enhance *Campylobacter* species infection to dog owners and their dogs		
Yes	117	62.2
No	11	5.9
I do not know	60	31.9

Multivariable analysis using a binary logistic regression model revealed that SHWs who attained tertiary education had slightly higher odds (OR = 1.4; *p* < 0.001) of obtaining a higher knowledge score with respect to the modes of transmission of *Campylobacter* infections compared to those without tertiary education (Table 5).

**Table 5:** Association between socioeconomic variables and knowledge of modes of *Campylobacter* species infections among carcass processors (*n* = 188) surveyed in Enugu State, Nigeria.

Variable	Category	OR (95% CI)	p-value
Gender	Male	1 (Ref)	–
	Female	1.024 (0.319–3.283)	0.968
Age category	< 45 years	1 (Ref)	–
	≥ 45 years	1.037 (0.521–2.063)	0.918
Marital status	Single	1 (Ref)	–
	Married	0.990 (0.497–1.973)	0.977
Occupation	Poultry carcass processor	1 (Ref)	–
	Cattle carcass processor	1.075 (0.539–2.143)	0.837
Location	Nsukka (peri-urban/rural)	1 (Ref)	–
	Enugu (urban)	0.954 (0.488–1.866)	0.891
Work experience	< 10 years	1 (Ref)	–
	≥ 10 years	1.168 (0.590–2.311)	0.655
Training on hygienic carcass processing	Yes	1 (Ref)	–
	No	1.486 (0.685–3.222)	0.316
Education	Tertiary education	1 (Ref)	–
	No tertiary education	1.37 (0.2751.814	< 0.001*

OR = Odds Ratio; CI = Confidence Interval; Ref = Reference category, *p < 0.05 indicates statistically significant association.

### 3.4 Manifestation of symptoms associated with human campylobacteriosis

Most of respondents (64.4%, 121/188) reported experiencing gastroenteritis symptoms (including diarrhoea, dysentery, and abdominal cramps) that align with campylobacteriosis within the 90 days preceding the survey. The majority (76%; 92/121) of those affected continued to report to work and were actively involved in processing carcasses and meat whilst exhibiting symptoms of the disease. Multivariable logistic regression analysis identified several factors that were significantly associated with gastroenteritis symptoms suggestive of campylobacteriosis among SHWs, and these included gender (*p* = 0.032), slaughterhouse location (*p* = 0.004), work experience (*p* = 0.006), prior training on hygienic carcass processing methods (*p* = 0.027), knowledge category (*p* < 0.001), and risk category (p < 0.001) (Table 6).

**Table 6:** Multivariable logistic regression analysis of factors associated with self-reported gastroenteritis symptoms among slaughterhouse workers (*n* = 188).

Predictor Variable	Category	AOR	95% CI (Lower–Upper)	p-value
Gender	Male	1 (Ref)	–	–
	Female	7.049	1.185–41.925	0.032*
Age group	< 45 years	1 (Ref)	–	–
	≥ 45 years	1.135	0.543–2.371	0.736
Occupation	Poultry processor	1 (Ref)	–	–
	Beef processor	0.970	0.466–2.020	0.936
Location	Nsukka (peri-urban/rural)	1 (Ref)	–	–
	Enugu (urban)	3.012	1.420–6.391	0.004*
Work experience	< 10 years	1 (Ref)	–	–
	≥ 10 years	2.841	1.357–5.945	0.006*
Training on hygienic processing	Yes	1 (Ref)	–	–
	No	2.739	1.124–6.670	0.027*
Educational level	Tertiary	1 (Ref)	–	–
	No tertiary	1.910	0.753–4.841	0.173
Knowledge category	High knowledge	1 (Ref)	–	–
	Low knowledge	4.464	2.021–9.865	<0.001*
Risk category	Low risk	1 (Ref)	–	–
	High risk	6.976	3.000–16.221	<0.001*
PPE use	Yes	1 (Ref)	–	–
	No	2.87	1.45–5.69	0.002*
Hand washing after slaughter operations	Yes	1 (Ref)	–	–
	No	3.14	1.62–6.09	<0.001*

Ref = Reference category; AOR = Adjusted Odds Ratio; CI = Confidence Interval; PPE = Personal Protective Equipment; Significant at *p* < 0.05.

The odds of female SHWs exhibiting clinical signs consistent with campylobacteriosis were 7 times (AOR = 7.049; *p* = 0.032) higher compared to their male counterparts (Table 6). Likewise, SHWs in Enugu were 3 times more likely to report such symptoms than those in Nsukka (Table 6). Workers with <10 years of occupational experience had 2.8-fold odds of manifesting symptoms compared to those with ≥10 years of experience. Furthermore, SHWs without tertiary education were twice (AOR = **1.9;** *p* **=** 0.173**)** as likely to experience gastroenteritis symptoms as compared to SHWs who attained tertiary education. The SHWs with a low knowledge score, were 5 times (AOR **=** 4.464; *p* ≤0.001) more likely to report symptoms of gastroenteritis compared to those who had high knowledge of *Campylobacter* transmission. Similarly, workers who were categorized as low risk workers were 7 times (AOR **=** 6.976) more likely to report symptoms associated with campylobacteriosis compared to the high-risk category of workers (Table 6).

## 4 Discussion

This study showed that over half of the SHWs were classified as being at high-risk of *Campylobacter* infection, based on a mean risk score of 2.8 ± 1.2. High-risk practices like non-use of personal protective equipment (77.1%), washing multiple carcasses in the same water bowl (54.8%), eating or drinking during carcass processing (45.7%), and consumption of raw or undercooked meat (36.7%), found in the study could collectively provide multiple pathways for zoonotic transmission of *Campylobacter* from animal reservoirs to humans during slaughterhouse operations. The preponderance of these risk behaviours is not surprising, given that 75% of respondents lacked formal training in hygienic meat processing. In the informal meat and food processing sector in Africa, particularly at the slaughterhouse level, untrained and minimally educated personnel constitute a critical food safety hazard, often facilitating the release or sale of meat contaminated with zoonotic bacteria, thereby exposing unsuspecting consumers to infection [43], [44], [45], [46], [47]. Similarly, unsafe carcass handling practices have been reported in abattoirs in Ghana and Ethiopia, where neglect of the use of PPE and improper carcass washing were directly linked to contamination events and foodborne disease outbreaks [48], [49]. Conversely, European studies underscore the effectiveness of strict regulatory enforcement of PPE usage and hygiene protocols in reducing occupational exposure and contamination risks [50]. These findings highlight the urgent need for enforceable occupational safety policies, provision of affordable PPE, and mandatory, routine supervision of slaughterhouse operations. Implementing these measures, particularly in low- and middle-income countries, is essential for interrupting zoonotic transmission of *Campylobacter* and safeguarding public health at the animal–human interface.

The reliance on non-potable water by 90.4% of SHWs for carcass processing has potential to significantly increase the risk of *Campylobacter* transmission. This is because water can serve as both a vehicle and an environmental reservoir for *Campylobacter*, facilitating pathogen dissemination to meat, consumers, and surrounding communities [51]. The absence of basic amenities such as potable water within slaughter facilities may have compelled the workers to use the same bowl of water for processing multiple carcasses, thereby increasing cross-contamination risks. Furthermore, borehole and well water, commonly utilized for processing, have been reported to contain coliforms exceeding 500 cfu/mL [52], highlighting the microbiological vulnerability of meat processed using water from such sources. Regional studies in Kenya and Tanzania similarly identified abattoir water as a major source of *Campylobacter* contamination in meat products [53], [54]. By contrast, high-income countries enforce strict regulations mandating potable water for carcass processing, which functions as a critical control point and substantially reduces environmental and consumer exposure to zoonotic pathogens [50], [55]. Addressing this infrastructural gap requires context-specific and One Health-based intervention that ensure sustainable access to potable water in slaughterhouses. Such measures would mitigate carcass contamination, limit environmental dissemination of *Campylobacter*, and enhance food safety and public health outcomes, thereby reinforcing integrated One Health strategies in LMICs.

The risk of *Campylobacter* infection associated with unsafe slaughterhouse practices is further exacerbated by poor knowledge regarding the modes of zoonotic transmission. The combination of limited understanding of *Campylobacter* transmission dynamics and engagement in high-risk behaviours likely increases the probability of infection among meat processors. This may explain why 64.4% of respondents reported gastroenteritis symptoms indicative of campylobacteriosis. While thorough cooking, a common cultural practice in many parts of Africa, can inactivate most zoonotic pathogens [56], [57], behaviours such as consumption of raw or undercooked meat, non-use of PPE, and eating or drinking during carcass processing substantially elevate the risk of zoonotic pathogen exposure. Infection among carcass processors poses a significant public health concern, as symptomatic workers who continue processing meat can serve as direct vectors, contaminating products destined for human consumption [58], [59], [60]. Specific knowledge gaps identified in this study, including not being aware that consumption of raw meat, use of contaminated water, non-use of PPE, and eating during carcass handling, reflect similar findings in Tanzania and Uganda, where abattoir workers demonstrated low awareness of zoonotic pathogens and engaged in unsafe handling practices [61], [62]. By contrast, European abattoirs report higher levels of awareness, attributable to structured training programs and continuous professional education [63]. Context-specific, low-literacy training modules that emphasize zoonotic pathogen transmission pathways should be integrated into occupational programs for SHWs in LMICs, representing a cost-effective One Health intervention to address critical knowledge deficits and reduce occupational and public health risks.

The finding that SHWs with tertiary education had slightly higher odds of possessing inadequate knowledge compared to their no tertiary-educated counterparts highlights the critical role of formal education in enhancing comprehension of microbial hazards and adoption of safer practices. Similar associations have been reported in Cameroon, where limited educational attainment was a strong predictor of unsafe food-handling behaviours [64]. Nevertheless, reliance on formal education alone is insufficient to guarantee improved practices, particularly in developing countries where the majority of SHWs have limited or no access to higher education. This underscores the need for inclusive and sustainable interventions that transcend formal education barriers. Beyond advocating for improved access to education, governments, regulatory authorities, and stakeholders must prioritize compulsory, practical, and context-specific training sessions on zoonotic disease risks and hygienic carcass processing within abattoirs. Such interventions, delivered in local languages and adapted to low-literacy settings, would ensure inclusivity while strengthening occupational safety, food hygiene, and public health protection.

The high prevalence of gastroenteritis symptoms among SHWs suggests compelling evidence of occupational vulnerability to *Campylobacter* infections. Specifically, 64.4% of respondents reported symptoms consistent with campylobacteriosis within the 90 days preceding the survey, of which 76% continued to process carcasses while the symptoms lasted. This practice creates a direct pathway for zoonotic transmission, not only through on-going occupational exposure to co-workers but also through contamination of carcasses and meat products intended for human consumption [58], [59], [60]. The continuation of work by symptomatic individuals underscores a dangerous gap in occupational health governance, where economic necessity and lack of institutionalized safeguards permit the perpetuation of infection cycles. Such patterns have similarly been documented in other countries, where symptomatic food handlers and butchers often remain at work due to the absence of labour protections or sick-leave allowances, thereby heightening the risk of foodborne outbreaks and community-level dissemination [65]. In contrast, high-income countries enforce strict exclusion policies for symptomatic food handlers, which serve as a protective barrier against zoonotic transmission and food contamination [59].

The study further revealed that SHWs with ten or more years of experience had higher odds of exhibiting symptoms compared to their less experienced counterparts. This finding suggests that prolonged occupational exposure in unsanitary environments not only accumulates health risks but may also entrench unsafe carcass handling practices over time. The absence of regular refresher training exacerbates this vulnerability by allowing knowledge gaps and risky activities to persist uncorrected. Evidence from Ethiopia and Bangladesh demonstrates that structured hygiene and biosafety training programs significantly reduce microbial contamination within abattoirs and lower the prevalence of occupational illness among workers [61], [62]. These findings suggest that targeted training interventions, particularly when delivered periodically and tailored to the literacy levels of the meat processors, can be highly effective preventive strategies.

## 5 Limitations of the study

As with all questionnaire based studies, potential recall and social desirability biases may have led to underreporting of risky behaviours or overestimation of knowledge. This study is limited by reliance on self-reported gastroenteritis without laboratory confirmation, which may underestimate or misclassify the true burden of *Campylobacter* infection, as other pathogens cause similar symptoms. Also, the study's restriction to two senatorial zones in one state limits broader (national or continental) generalizability. Nevertheless, the findings remain robust and provide valuable insights that contribute meaningfully to global public health improvement, particularly in resource-limited settings of low-and medium-income countries.

## 6 Conclusion

Unsafe practices, including non-use of PPE, consumption of non-potable water, and eating during carcass processing, place slaughterhouse workers at high risk of *Campylobacter* exposure. Knowledge gaps and low hygiene training further exacerbate occupational vulnerability. Low work experience (10 years, lack of training, low knowledge, and high-risk practices were independently associated with self-reported gastroenteritis symptoms. Targeted One Health interventions, including provision of potable water, mandatory use of PPE, context-specific hygiene training, and strengthened regulatory oversight are urgently needed to reduce zoonotic *Campylobacter* transmission, protect workers, and improve food safety in Nigeria and other LMICs. Policymakers should enact occupational health regulations that provide economic and social protections for workers, including sick-leave allowances to discourage symptomatic individuals from continuing to process meat.

## CRediT authorship contribution statement

**Emmanuel O. Njoga:** Writing – review & editing, Writing – original draft, Validation, Resources, Methodology, Investigation, Formal analysis, Data curation, Conceptualization. **Kennedy F. Chah:** Writing – review & editing, Supervision, Resources. **James W. Oguttu:** Writing – review & editing, Supervision, Resources.

## Ethical approval

Ethical approval for this study was obtained from the Research Ethics Committee of the Enugu State Ministry of Health (Reference No. MH/MSD/REC21/232). Informed consent was requested for and obtained from all participants prior to the study.

## Funding

This research did not receive any specific grant from funding agencies in the public, commercial, or not-for-profit sectors.

## Declaration of competing interest

The authors declare that they have no known competing financial interests or personal relationships that could have appeared to influence the work reported in this paper.

## Data Availability

The datasets generated and/or analyzed during the current study are within the manuscript. Further details are available from the corresponding author on reasonable request.
